# Supplementary material for: ATG8-dependent LMX1B-autophagy crosstalk shapes human midbrain dopaminergic neuronal resilience
Source: J Cell Biol. 2023 Apr 4;222(5):e201910133. doi: 10.1083/jcb.201910133 (PMC10075225; doi:10.1083/jcb.201910133)
Supplement: Table S1 — shows MatInspector promoter FLAT sequence analysis for selected candidate gene. [file JCB_201910133_TableS1.docx]

**Table S1. MatInspector promoter FLAT sequence analysis for selected candidate genes**

|  | Gene symbol | Putative LMX1A Binding site | Position relative to TSS | Score (0-1) | Putative LMX1B Binding site | Position relative to TSS | Score (0-1) |
| --- | --- | --- | --- | --- | --- | --- | --- |
| Transcription factors | **TFEB** | agtgcaggttAATTactgcccag *§*  gctgggcagtAATTaacctgcac *§* | -297  -298 | 0.878  0.876 | gggaagtgcaggtTAATtactgc *§*  agtgcaggttAATTactgcccag *§*  gctgggcagtAATTaacctgcac *§* | -293  -297  -298 | 0.938  0.878  0.87 |
|  | **ZKSCAN3** | ttcatattctAATTaggcaatgc  tgcattgcctAATTagaatatga | -124  -125 | 0.839  0.861 | ttcatattctAATTaggcaatgc  tgcattgcctAATTagaatatga  aaaatcggaaaatTAATaatggt | -124  -125  -258 | 0.851  0.832  0.971 |
|  | **NRF1** |  | | | ttattaatttTATTaactcttct  gagttaataaaatTAATaatatt  gcacattcttAAATaaaatggac  agtccattttATTTaagaatgtg  atatttgtttAATGaatgtggta | -654  -658  -693  -694  -1082 | 0.844  0.963  0.837  0.84  0.844 |
|  | **NRF2** |  | | | attatttaaaaatTAATcattgc  attaatttttAAATaattgtttt  taacaattttAATAaatctgttt | -330  -336  -603 | 0.938  0.865  0.857 |
| Autophagy genes | **ULK1** |  | | | caaagattatAATTaagtaaaag *§* | -153 | 0.909 |
|  | **ULK2** | tgatacagttAATTgaaaagtta  taaaagtattAATAaaaagaact  aagttcttttTATTaatactttt  acttctgtttAATTtatttcaaa  atttgaaataAATTaaacagaag  aatcctttttGATTaatatgaac  agttcatattAATCaaaaaggat  gaatcaacttTATTaatacaaaa  gaatggattgAATTaatcttgaa  attcaagattAATTcaatccatt  cttattctttAATTgaattttta  ataaaaattcAATTaaagaataa  tatcaaggctAATTaagagattt | -196  -537  -538  -165  -166  -235  -236  -286  -422  -423  -367  -368  -216 | 0.869  0.852  0.851  0.843  0.856  0.842  0.862  0.845  0.836  0.846  0.853  0.856  0.887 | ataacttttcAATTaactgtatc  taaaagtattAATAaaaagaact  aagttcttttTATTaatactttt  acttctgtttAATTtatttcaaa  atttgaaataAATTaaacagaag  aatcctttttGATTaatatgaac  agttcatattAATCaaaaaggat  tttttgtattAATAaagttgatt  gaatcaacttTATTaatacaaaa  gaatggattgAATTaatcttgaa  attcaagattAATTcaatccatt  cttattctttAATTgaattttta  ataaaaattcAATTaaagaataa | -195  -537  -538  -165  -166  -235  -236  -285  -286  -422  -423  -367  -368 | 0.856  0.836  0.852  0.858  0.844  0.862  0.839  0.84  0.834  0.856  0.836  0.849  0.85 |
|  | **ATG2A** |  | | | | | |
|  | **ATG2B** | ctagtttatcAATTaacaaagta  gtactttgttAATTgataaacta  tagcagatttATTTaaagtaaga  agttatttttAATTaggtttttt  caaaaaacctAATTaaaaataac  cactacaaatAATTataatgcaa  aaatggatttATTTaaatacttt  aaaagtatttAAATaaatccatt  agaaatgcttAATTaactccaaa  atttggagttAATTaagcatttc  ctccaaatttAATTactgttttg  ccaaaacagtAATTaaatttgga | -14  -15  -569  -405  -406  -28  -350  -351  -395  -396  -411  -412 | 0.85  0.859  0.837  0.894  0.928  0.867  0.854  0.838  0.932  0.947  0.893  0.905 | ctagtttatcAATTaacaaagta  gtactttgttAATTgataaacta  ttcttctgaatatTAATcatgga  atcttactttAAATaaatctgct  agttatttttAATTaggtttttt  caaaaaacctAATTaaaaataac  attgcattatAATTatttgtagt  agagcactacaaaTAATtataat  aaatggatttATTTaaatacttt  aaaagtatttAAATaaatccatt  catgaatcttATTTaatgtaaat  agaaatgcttAATTaactccaaa  atttggagttAATTaagcatttc  ctccaaatttAATTactgttttg  ccaaaacagtAATTaaatttgga | -14  -15  -482  -568  -405  -406  -27  -32  -350  -351  -396  -395  -396  -411  -412 | 0.852  0.854  0.915  0.832  0.925  0.895  0.929  0.947  0.845  0.855  0.841  0.943  0.938  0.967  0.942 |
| Autophagy genes | **ATG3** | ggtactggttAATTagttatatt *§*  aaatataactAATTaaccagtac *§* | -555  -556 | 0.902  0.899 | ggtactggttAATTagttatatt *§*  aaatataactAATTaaccagtac *§* | -555  -556 | 0.898  0.901 |
|  | **ATG4A** |  | | | | | |
|  | **ATG4B** | aaaactcttgAATTaagtatgca  atgcatacttAATTcaagagttt | -333  -334 | 0.833  0.843 | aaaactcttgAATTaagtatgca  atgcatacttAATTcaagagttt | -333  -334 | 0.836  0.834 |
|  | **ATG4C** | tgtgacttgtAATTagattatga  ctcataatctAATTacaagtcac  ttccttcttaAATTaaatattta  ctaaatatttAATTtaagaagga | -559  -560  -391  -392 | 0.852  0.862  0.861  0.851 | tgtgacttgtAATTagattatga  ctcataatctAATTacaagtcac  ttccttcttaAATTaaatattta  ctaaatatttAATTtaagaagga | -559  -560  -391  -392 | 0.848  0.84  0.861  0.853 |
|  | **ATG4D *** |  | | | | | |
|  | **ATG5** | actaatgtttTATTaatactgtt  caacagtattAATAaaacattag  gaagtacagtAATTaaaatggaa  cttccattttAATTactgtactt | -281  -282  -28  -29 | 0.844  0.854  0.912  0.887 | actaatgtttTATTaatactgtt  caacagtattAATAaaacattag  aaaggaagtacagTAATtaaaat  gaagtacagtAATTaaaatggaa  cttccattttAATTactgtactt  aatacttccatttTAATtactgt | -281  -282  -24  -28  -29  -33 | 0.847  0.833  0.935  0.882  0.912  0.929 |
|  | **BECLIN1 *** | gtagtttgttCATTaaacaaaat  gattttgtttAATGaacaaacta  gaagtgacgtAATTacacttgta | -312  -313  -415 | 0.834  0.841  0.831 |  | | |
|  | **ATG7** | attcctcattTATTaatcagagc *§*  agctctgattAATAaatgaggaa *§* | -212  -213 | 0.839  0.835 | attcctcattTATTaatcagagc *§*  agctctgattAATAaatgaggaa *§* | -212  -213 | 0.83  0.835 |
|  | **GABARAPL1*** |  | | | | | |
|  | **GABARAPL2** |  | | | | | |
|  | **ATG9A** |  | | | | | |
|  | **ATG9B*** |  | | | | | |
|  | **ATG10** | tgcagagtttTATTaaaaaatta  ataattttttAATAaaactctgc  cccaggtgctAATTaaaagtaat  aattacttttAATTagcacctgg  aattaaaagtAATTattttgggt  gacccaaaatAATTacttttaat  taagtgattaAATTaatctgaat  gattcagattAATTtaatcactt  gtgcagaattCATTaaacattca  ttctgccttaAATTaaaacaaag  actttgttttAATTtaaggcaga | -324  -325  -431  -432  -441  -442  -580  -581  -571  -316  -317 | 0.867  0.845  0.906  0.902  0.843  0.845  0.837  0.851  0.855  0.854  0.832 | tgcagagtttTATTaaaaaatta  ataattttttAATAaaactctgc  cccaggtgctAATTaaaagtaat  aattacttttAATTagcacctgg  tgctaattaaaagTAATtatttt  aattaaaagtAATTattttgggt  gacccaaaatAATTacttttaat  taagtgattaAATTaatctgaat  gattcagattAATTtaatcactt  gtgaatgtttAATGaattctgca  ttctgccttaAATTaaaacaaag  actttgttttAATTtaaggcaga  tctttatcttAACTaattgggaa | -324  -325  -431  -432  -437  -441  -442  -580  -581  -572  -316  -317  -582 | 0.836  0.85  0.895  0.906  0.955  0.843  0.989  0.853  0.835  0.85  0.833  0.845  0.841 |
| Autophagy genes | **ATG12 *** |  | | | | | |
|  | **ATG13** | ttttctttttAATTtaatttagt  aactaaattaAATTaaaaagaaa  tttttaatttAATTtagttttct  aagaaaactaAATTaaattaaaa  aaggaatataAATTaactaatgt  cacattagttAATTtatattcct  ttgaatggatAATTaaagctcaa  tttgagctttAATTatccattca | -62  -63  -67  -68  -489  -490  -576  -577 | 0.85  0.872  0.839  0.848  0.843  0.857  0.884  0.885 | ttttctttttAATTtaatttagt  aactaaattaAATTaaaaagaaa  tttttaatttAATTtagttttct  aagaaaactaAATTaaattaaaa  aaggaatataAATTaactaatgt  cacattagttAATTtatattcct  aatataaattAACTaatgtgtgt  aacacacattAGTTaatttatat  ttgaatggatAATTaaagctcaa  tttgagctttAATTatccattca | -62  -63  -67  -68  -489  -490  -493  -494  -576  -577 | 0.865  0.852  0.854  0.841  0.851  0.85  0.845  0.836  0.87  0.87 |
|  | **ATG14** | gaacatttttAATTacttctgtt  gaacagaagtAATTaaaaatgtt | -469  -470 | 0.892  0.931 | gaacatttttAATTacttctgtt  gaacagaagtAATTaaaaatgtt | -469  -470 | 0.925  0.921 |
|  | **ATG16L1** | tacttgactgAATTaaatgttaa *§*  cctaaaaagtAATTagatatgtc *§*  tgacatatctAATTactttttag *§* | -82  -146  -147 | 0.841  867  0.852 | gttaacatttAATTcagtcaagt *§*  cctaaaaagtAATTagatatgtc *§*  tgacatatctAATTactttttag *§* | -83  -146  -147 | 0.842  0.935  0.865 |
|  | **ATG16L2** |  | | | | | |
|  | **ATG17** | tttccgagttAATTagtagacat  catgtctactAATTaactcggaa  cttaaagctgAATTaaacaaagt | -331  -331  -372 | 0.908  0.885  0.835 | tttccgagttAATTagtagacat  catgtctactAATTaactcggaa  tactttgtttAATTcagctttaa | -331  -332  -373 | 0.893  0.904  0.833 |
|  | **WIPI1** |  | | | | | |
|  | **WIPI2** | acatctttttAATAaatatgtgt  gacacatattTATTaaaaagatg  tttgtgtcttAATTaagggagcc  gggctcccttAATTaagacacaa | -368  -369  -507  -508 | 0.852  0.867  0.916  0.946 | acatctttttAATAaatatgtgt  gacacatattTATTaaaaagatg  tttgtgtcttAATTaagggagcc  gggctcccttAATTaagacacaa | -368  -369  -507  -508 | 0.864  0.841  0.94  0.924 |
|  | **ATG101** |  | | | | | |
|  | **mTOR** | tagagaatctAATTaaaaacatt | -509 | 0.938 | aaatgtttttAATTagattctct  tctctacaaaaaaTAATtagcca  tgctgctgtagaaTAATaatctc  gtcattgtttAATGaatctaggc | -510  -178  -192  -834 | 0.933  0.961  0.938  0.842 |
| Autophagy genes | **AMBRA1** | gaaggggattAATGaagagccac | -569 | 0.833 | ggtggctcttCATTaatcccctt  aaatgaggttAACTaatttgctc  agaacttaaagtaTAATaataaa  acttaaagtataaTAATaaaata  atttatatataaaTAATaaaata  atatttatataaaTAATaaaata  atttatatataaaTAATaaaata  atttatatataaaTAATaaaaca  tattatatataaaTAATaaaata  atttatatataaaTAATaaaata | -570  -246  -77  -80  -128  -152  -178  -211  -234  -269 | 0.832  0.831  0.911  0.929  0.929  0.929  0.929  0.914  0.929  0.929 |
|  | **CALCOCO1** |  | | | aagtggcttaaatTAATtaggat  ggacagataacaaTAATaacgat | -378  -917 | 0.97  0.958 |
|  | **PIK3C3*** |  |  |  | tttggccatgAATTaatgcataa  tctccaagtgAATTaagtttcct  aaggaaacttAATTcacttggag | -404  -31  -32 | 0.834  0.83  0.835 |
|  | **PIK3R4** | actgtcaattAAATaaaaaaaaa  ttttttatttAATTgacagtttg  tcaaactgtcAATTaaataaaaa  aagattatatAATTacagtctga  atcagactgtAATTatataatct  ggatagaatgAATTaatatatgt  tacatatattAATTcattctatc  aaaaaattttAATTactgtgtta  ttaacacagtAATTaaaattttt | -431  -434  -435  -534  -535  -81  -82  -404  -405 | 0.86  0.841  0.851  0.844  0.853  0.861  0.838  0.885  0.919 | ttagaaagaaaagTAATaaattg  caacaaataaaatTAATaagaaa  ctttttttttATTTaattgacag  ttttttatttAATTgacagtttg  tcaaactgtcAATTaaataaaaa  aagattatatAATTacagtctga  ggatagaatgAATTaatatatgt  tacatatattAATTcattctatc  aaagtttaagaaaTAATaaaatt  aaaaaattttAATTactgtgtta  ttaacacagtAATTaaaattttt | -169  -218  -430  -434  -435  -534  -81  -82  -149  -404  -405 | 0.93  0.947  0.864  0.849  0.836  0.914  0.852  0.838  0.929  0.936  0.931 |
|  | **LC3A** | acttaggcctAATTaactgaagt  aacttcagttAATTaggcctaag  acccccagctAATTattgtattt | -512  -513  -169 | 0.87  0.875  0.834 | acttaggcctAATTaactgaagt  aacttcagttAATTaggcctaag  actaaaaatacaaTAATtagctg | -512  -513  -164 | 0.874  0.875  0.956 |
|  | **LC3B*** |  |  |  |  |  |  |
|  | **LC3C** |  |  |  |  |  |  |
|  | **LAMP1** |  |  |  |  |  |  |
|  | **LAMP2** | atgcccagctAATTattatcttt | -103 | 0.86 | aaaagataatAATTagctgggca  tctactaaaaagaTAATaattag | -104  -111 | 0.939  0.933 |
|  | **UVRAG** | atatgattttCATTaaaaggatg *§*  ccatccttttAATGaaaatcata *§*  tggtgctggtAATTaagttactc *§*  tgagtaacttAATTaccagcacc *§*  gtgagtatgtAATTattcatagt *§*  gactatgaatAATTacatactca *§* | -288  -289  -309  -310  -394  -395 | 0.842  0.855  0.879  0.885  0.842  0.86 | gatttttaaaactTAATaataag  atatgattttCATTaaaaggatg *§*  ccatccttttAATGaaaatcata *§*  tggtgctggtAATTaagttactc *§*  tgagtaacttAATTaccagcacc *§*  ctcttgagtaactTAATtaccag *§*  gtgagtatgtAATTattcatagt *§*  tcatgactatgaaTAATtacata *§* | -155  -288  -289  -309  -310  -314  -394 | 0.918  0.849  0.84  0.885  0.874  0.941  0.844  0.941 |
| Mitophagy genes | **PARKIN** | ctaaagacttAATTaaatgagtc  tgactcatttAATTaagtcttta  ccaactccttGATTaaagagctc  ccagtttggtAATTattttttcc  aggaaaaaatAATTaccaaactg | -55  -56  -583  -225  -226 | 0.954  0.95  0.832  0.833  0.853 | aggactaaagactTAATtaaatg  ctaaagacttAATTaaatgagtc  tgactcatttAATTaagtcttta  ccagtttggtAATTattttttcc  aggaaaaaatAATTaccaaactg  ccaccaatcaaaaTAATaagtac | -51  -55  -56  -225  -226  -37 | 0.914  0.947  0.96  0.839  0.992  0.941 |
|  | **OPTINEURIN** | cactgcatttGATTaatgattta *§*  ataaatcattAATCaaatgcagt *§*  tgacggcgttAATCaaaataaat *§*  cactgcatttGATTaatgattta *§*  ataaatcattAATCaaatgcagt *§*  tgacggcgttAATCaaaataaat *§*  acacctggctAATTattaaattt | -289  -290  -307  -915  -916  -933  -288 | 0.841  0.839  0.843  0.841  0.839  0.843  0.857 | cactgcatttGATTaatgattta *§*  gatttattttGATTaacgccgtc *§*  cactgcatttGATTaatgattta *§*  gatttattttGATTaacgccgtc *§*  aaaatttaatAATTagccaggtg  tttacaaaaaattTAATaattag  aaaatttaatAATTagccaggtg | -289  -306  -915  -932  -289  -296  -289 | 0.846  0.84  0.846  0.84  0.837  0.933  0.837 |
|  | **PINK1** | tggtgcagatAATTatttcctta  ctgttaaataAATTaaaagacgt *§*  tacgtcttttAATTtatttaaca *§* | -32  -370  -371 | 0.84  0.865  0.841 | tattttaaggaaaTAATtatctg  tggagctgttAAATaaattaaaa *§*  cttttaatttATTTaacagctcc *§*  ctgttaaataAATTaaaagacgt *§*  tacgtcttttAATTtatttaaca *§* | -27  -365  -366  -370  -371 | 0.947  0.832  0.835  0.843  0.866 |
|  | **NDP52** | aagtcactttAATTtagcaccct *§* | -633 | 0.835 | acaactacaacagTAATaactat *§* | -289 | 0.921 |
|  | **P62** |  | | | | | |
|  | **NBR1** |  | | | ccacagttttAATTtatctgtaa  ccacagttttAATTtatctgtaa | -253  -752 | 0.859  0.859 |
|  | **BNIP3L1** | aacagggtttAATGaaaacccta | -1097 | 0.854 | ctagggttttCATTaaaccctgt | -1907 | 0.841 |
|  | **FUNDC1** |  | | | ttttaaaacaaaaTAATcaaatt | -862 | 0.923 |
|  | **TAX1BP1** | ttcaagaaatAATTaaaaataac  cagtatgcttAATAaaaaccata  cagtatgcttAATAaaaaccata  ctgtaagcttAATTacaagaaag | -223  -508  -237  -204 | 0.933  0.847  0.847  0.895 | tcgtatgtcaaaaTAATaacata  aaaattcaagaaaTAATtaaaaa  ttatggttttTATTaagcatact  ttatggttttTATTaagcatact  cataatgaaaaacTAATaaagta  tctttcttgtAATTaagcttaca  ataactgtaagctTAATtacaag  tgcttgattgAATTaaggaattt | -821  -219  -507  -236  -136  -203  -208  -877 | 0.952  0.958  0.838  0.838  0.926  0.889  0.929  0.836 |
| Mitochondrial genes | **NDUFA2** | cgtgttgtgtAATTacacattat | -15 | 0.843 | agactgattaAATTaacgtcaat | -339 | 0.84 |
|  | **NDUFA3** |  | | | | | |
|  | **NDUFA4 *** |  | | | | | |
|  | **NDUFV1** |  | | | caagagagcggaaTAATtatgaa | -541 | 0.938 |
|  | **UQCRQ** |  | | | tcagccacaaaaaTAATcaaata | -444 | 0.938 |
|  | **COX1** | caaggcatttTATTaattagtgt | -716 | 0.856 | ggcgagcatacacTAATtaataa | -707 | 0.935 |
| Known target genes | **NURR1** | ctgggaatatAATTaaatataaa | -355 | 0.919 | ctctcattttAATTtatatttaa  tgagagagagagaTAATtagaat  ttaactgggaataTAATtaaata  ctatattaaaaaaTAATaactgc *§*  taactctggacttTAATaaaggc  tgagagagagagaTAATtagaat | -331  -434  -359  -742  -107  -89 | 0.867  0.914  0.952  0.967  0.918  0.914 |
|  | **PITX3** | ctacattttTATTaattcaata *§*  ttattgaattAATAaaaatgtag *§*  catttttattAATTcaataaata *§*  atatttattgAATTaataaaaat *§* | -200  -201  -204  -205 | 0.835  0.868  0.857  0.862 | tctacattttTATTaattcaata *§*  ttattgaattAATAaaaatgtag *§*  catttttattAATTcaataaata *§*  atatttattgaatTAATaaaaat *§* | -200  -201  -204  -205 | 0.862  0.835  0.858  0.918 |
|  | **TH** | ttctggtgttCATTaaaagtgtg | -480 | 0.838 | gcacacttttAATGaacaccaga | -481 | 0.836 |
|  | **ABRA** |  | | | tgggactagaaagtaattagtct *§*  gatagtcaaagactaattacttt *§* | -359  -368 | 0.913  0.937 |
|  | **IFNB** |  | | | ctttacaaaatatTAATaataaa | -145 | 0.936 |
|  | **COL4A3 *** |  | | | cgagcctccaaatTAATcaaaac  tgtgtgttttGATTaatttggag | -241  -246 | 0.927  0.859 |
| Negative control | **GAPDH** |  | | | | | |

* *LMX1A and/or LMX1B binding sites were only found in promoters for non-coding transcripts*

*§* *LMX1A/B binding sequences amplified with primers designed for ChIP assay*
